# Skin Barrier and Dermal Edema Recovery Using Ideal‐Sized Chitosan After Microneedle Radiofrequency: A Split‐Face Case Report

**DOI:** 10.1111/jocd.71131

**Published:** 2026-08-17

**Authors:** Jin Ho Kim, Won Seok Choi

**Affiliations:** ^1^ V&Co Cooperation Seoul Korea; ^2^ V‐Plastic Surgery Daegu Korea

**Keywords:** chitosan, microneedle radiofrequency, trans‐epidermal water loss

To the Editor,

Microneedling fractional radiofrequency (MNRF) is widely used for skin rejuvenation, but transiently compromises the epidermal barrier [1], triggering inflammation, trans‐epidermal water loss (TEWL), erythema, and acute dermal edema [2, 3]. Ideal‐sized chitosan (ISC) featuring low‐molecular‐weight (< 600 Da) possesses anti‐inflammatory and wound‐healing properties, making it an effective post‐procedure agent [4]. We present a split‐face case report utilizing objective biophysical analysis to evaluate ISC efficacy following MNRF.

A 32‐year‐old male received MNRF (Potenza, Jeisys Medical Inc.; 600 shots, 400 mJ, 1.5 mm depth). Immediately post‐procedure, 1.5 cc of ISC (Arche, Doum Inc.) was applied to the left face, while the right face served as an untreated control with informed consent. The applied ISC was left to facilitate absorption for 12 h. The patient completely removed the residual ISC film with water after 12 h. All measurements from 12 h were taken on bare skin to eliminate the immediate physical occlusive effect of the film as a confounding factor. At 48 h, rescue ISC (1.5 cc) was applied to the right face. Skin parameters—stratum corneum hydration (SCH), TEWL, erythema—and dermal ultrasound scans (SKENA) were evaluated longitudinally over 72 h.

Immediately following MNRF, both sides demonstrated acute barrier interruption and inflammation, marked by decrease in SCH, increase in TEWL and erythema (Table 1). Moreover, elevation in low echogenic pixels (LEPs) indicated acute dermal edema (Figure 1a,b). ISC treated side exhibited measurable reduction in erythema and dermal edema (Figure 1c,d). Compared to control, ISC treated side demonstrated a faster decline in TEWL, reflecting accelerated epidermal barrier restoration (Figure 2).

**TABLE 1:** Quantitative shifts in skin biophysical properties at baseline, immediately post‐procedure, and 24 h post‐MNRF for the control versus ideal‐sized chitosan (ISC)‐treated sides.

	Stratum corneum hydration (μS)	TEWL (g/m^2^/h)	Erythema (a.u.)
Pre‐procedure	177.2	16.1	18.17
Post‐procedure	108.8	39.2	26.13
Post‐24 h (control)	166.5	20.6	20.51
Post‐24 h (ISC apply)	280.5	15.8	18.25

**FIGURE 1:**
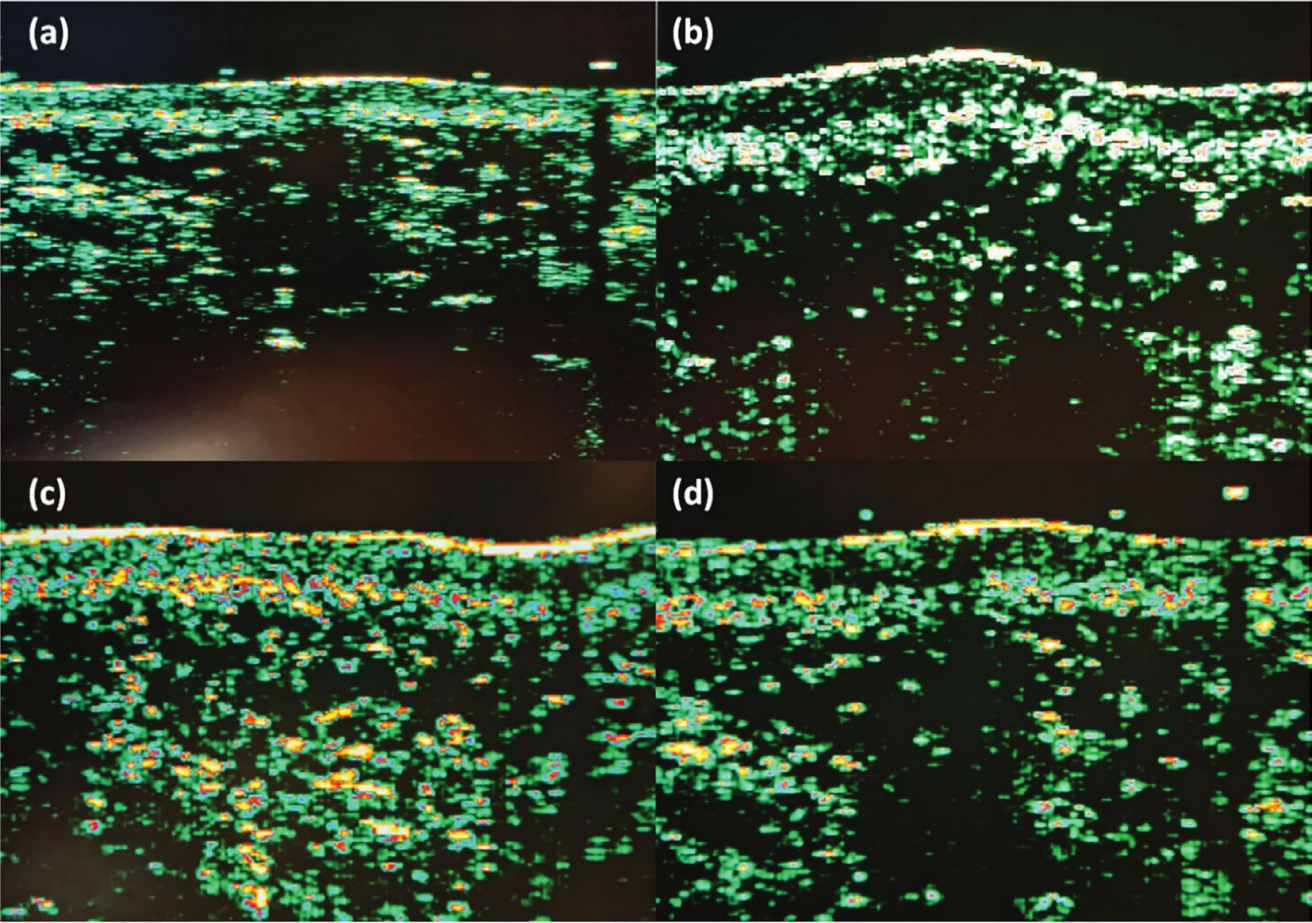
High‐resolution dermal ultrasound imaging demonstrating the evolution of acute dermal edema. (a) Pre‐procedure baseline showing normal dermal echogenicity. (b) Immediately post‐procedure, demonstrating an expansion of low echogenic pixels (LEP) due to acute inflammatory interstitial fluid exudation following MNRF. (c) At 24 h post‐procedure on the ISC‐applied side, showing resolution of edema and recovery of dermal density. (d) At 24 h post‐procedure on the untreated control side, exhibiting persistent edema and a high proportion of LEPs.

**FIGURE 2:**
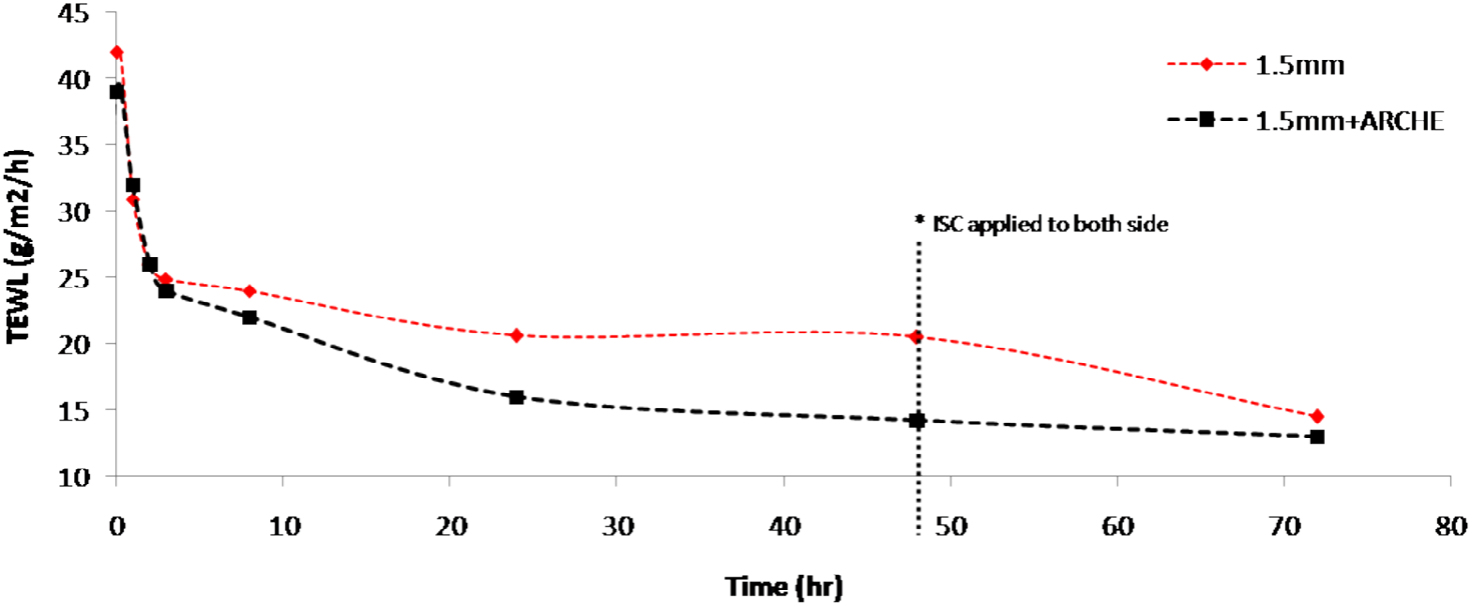
Longitudinal recovery kinetics of TEWL over 72 h. The curves highlight the immediate therapeutic acceleration on the early‐treated left face and the recovery trajectory observed on the right face immediately following delayed ISC rescue application at 48 h.

While the untreated side showed prolonged barrier impairment and delayed edema up to 48 h, the application of ISC at 48 h was followed by an accelerated recovery. Although rapid resolution could be coincided with the natural healing pathways after MNRF, the observed change in the recovery trajectory suggests a potential supportive therapeutic effect of the delayed ISC rescue application.

The observed acceleration of TEWL and dermal edema resolution on the ISC‐treated side may be attributed to the specific physical and biochemical properties of ultra‐low‐molecular‐weight chitosan. Recent biomaterial analysis confirmed that ISC exhibits a molecular weight distribution predominantly below 600 Da, which significantly enhances percutaneous absorption and targeting deeper layers to reduce acute post‐procedural inflammation [4]. Deep delivery directly aligns with our ultrasound findings showing prompt LEP resolution [2]. Additionally, immediate post‐procedure ISC application is clinically safe and feasible; a recent retrospective cohort of 324 patients reported an extremely low folliculitis incidence after treatments [5], supporting the complication‐free recovery observed in our patient [1, 5].

A key consideration is the confounding effect of physical occlusion during the initial phase [1]. Because the ISC remained on the skin for 12 h, the initial rapid decline in TEWL reflects a combined result of physical semi‐occlusion and biological healing [1, 3]. However, after the film was completely washed off at 12 h, TEWL did not rebound but continued to decline, accompanied by resolution of dermal edema [1, 2, 3]. This trajectory could indicate that the ISC induced biological restoration of the barrier and exerted anti‐inflammatory effects beyond mere physical surface occlusion [1, 2, 3]. Furthermore, because rescue therapy was applied to the control side at 48 h, the pure untreated control was lost beyond this point [1, 3]. While this limits our ability to completely isolate late‐stage ISC efficacy from the natural kinetics of barrier healing, the acceleration in recovery post‐application suggests a supportive therapeutic role even in late‐phase recovery [1, 3].

The primary limitations of this study are its single‐case design and the short‐term 72‐h follow‐up period. Larger randomized controlled trials are warranted to confirm these findings and investigate long‐term clinical benefits. In conclusion, this report suggests that the topical application of ISC may support epidermal barrier recovery and alleviate acute dermal edema following energy‐based devices.

## Author Contributions

Dr. Jin Ho Kim contributed to data curation, analysis, investigation, and writing original draft. Dr. Won Seok Choi contributed to conceptualization, validation, and reviewing original draft.

## Ethics Statement

Participant provided written informed consent.

## Conflicts of Interest

The authors declare no conflicts of interest.

## Data Availability

The data that support the findings of this study are available from the corresponding author, AC upon reasonable request.
